# PRevention of INCisional hernia after liver transplantation (PRINC trial): study protocol for a randomized controlled trial

**DOI:** 10.1186/s13063-019-3477-2

**Published:** 2019-06-20

**Authors:** Daniela Kniepeiss, James Elvis Waha, Thomas Auer, Andrea Berghold, Peter Schemmer

**Affiliations:** 1 General, Visceral and Transplant Surgery, Transplant Center Graz, Medical University of Graz, Graz, Austria; 2 Transplant Center Graz, Medical University of Graz, Graz, Austria; 3 General, Visceral and Transplant Surgery, Medical University of Graz, Graz, Austria; 4 Institute for Medical Informatics, Statistics and Documentation, Medical University of Graz, Graz, Austria; 5 Department of General, Visceral and Transplant Surgery, Transplant Center Graz, Medical University of Graz, Auenbruggerplatz 29, 8036 Graz, Austria

**Keywords:** Incisional hernia, Liver transplantation, Prophylactic mesh, Long-term absorbable mesh

## Abstract

**Background:**

Incisional hernia is a common complication after liver transplantation with an incidence of 5 to 46%. Concerning non-transplant patients, a recently published meta-analysis describes a reduction of the incidence of incisional hernia of up to 85% due to prophylactic mesh replacement in elective, midline laparotomy. The aim of our study is to show a reduction of the incidence of incisional hernia after liver transplantation with minimal risk for complication.

**Methods/design:**

This is an unblinded, randomized controlled trial comparing time to incisional hernia over a period of 12 months between patients undergoing liver transplantation and standardized abdominal closure with or without prophylactic placement of Phasix™ (Bard – Davol Inc., Warwick, RI, USA) mesh in an onlay position. As we believe that the mesh intervention is superior to the standard procedure in reducing the incidence of hernia, this is a superiority trial.

**Discussion:**

The high risk for developing incisional hernia following liver transplantation might be reduced by prophylactic mesh placement. Immunosuppressed patients are at high risk for developing surgical-site infections. We chose a mesh which has anti-inflammatory properties and is fully resorbed after 18 months.

**Trial registration:**

ClinicalTrials.gov, ID: 03222102. Registered retrospectively on 17 July 2018. Protocol version 1.4, 7 October 2018.

**Electronic supplementary material:**

The online version of this article (10.1186/s13063-019-3477-2) contains supplementary material, which is available to authorized users.

## Background

Incisional hernia is a common complication after liver transplantation with an incidence of 5 to 46% [1–12]. The high incidence rate is due to the very reduced general condition of the patients at the time of operation and is also dependent on the surgical technique, the materials used and the experience of the surgeon [13]. The essential immunosuppressive therapy after transplantation has a negative impact on wound healing and leads to the formation of dysfunctional scar tissue.

Artificial mesh is commonly used in ventral incisional hernia repair as described in the literature, but there is yet no scientific paper on the use of prophylactic mesh placement at the time of transplantation. The main reason, therefore, is that immunosuppressive therapy and artificial mesh implantation seem to be contradictory due an increased risk of complications.

Concerning non-transplant patients, a recently published meta-analysis by Borab et al. describes a reduction of the incidence of incisional hernia of up to 85% due to prophylactic mesh replacement in elective, midline laparotomy [14].

The development of Phasix™ (Bard – Davol Inc., Warwick, RI, USA), an absorbable mesh, has given reason to conduct a study aiming at improving the healing process and stability of the abdominal wall. The mesh consists of suture material, which has proven reliability for many years and is characterized by an extremely long absorption rate of 12 to 18 months.

Phasix™ (Bard – Davol Inc., Warwick, RI, USA) is a fully resorbable monofilament mesh consisting of poly-4-hydroxybutyrate (P4HB) (CE-certified) and is licensed for the treatment of hernias. P4HB is degraded by hydrolysis to the monomer 4-hydroxybutyrate (4-HB) which is a naturally occurring metabolite in humans. P4HB has been in clinical use since 2007 as the suture material Monomax® (Braun). Phasix™ (Bard – Davol Inc., Warwick, RI, USA) is comparable in repair strength to non-resorbable conventional mesh. It resorbs after 12 to 18 months and is gradually replaced by the host’s own tissue [15].

In comparison to other fully resorbable meshes, Phasix™ (Bard – Davol Inc., Warwick, RI, USA) shows higher stability over an even longer period. Particularly with regard to the stability during the first few weeks the material is strong enough to bridge the initial phase of healing. The phase of degradation is constant and restricted [16–20].

Preliminary results of a study conducted on rats at the University of Tennessee Health Science Center, Memphis, TN, USA showed a distinct decrease of methicillin-resistant *Staphylococcus aureus* (MRSA)-infected Phasix™ (Bard – Davol Inc., Warwick, RI, USA) mesh compared to other biological implants, which emphasizes its value for use on immunosuppressed patients even more [21].

## Methods/design

### Aim of the study

The aim of our study is to show a reduction in the incidence of incisional hernia after liver transplantation with minimal risks for complications. We believe that the mesh intervention is superior to the standard procedure in reducing the incidence of hernia; therefore, it is a superiority trial.

### Inclusion and exclusion criteria

Patients undergoing liver transplantation due to end-stage liver diseases at the Department of Surgery, Division of Transplantation Surgery, Medical University of Graz will be included after written informed consent has been obtained.

### Inclusion criteria

- Age > 18 years
- First liver transplantation
- Written informed consent provided

### Exclusion criteria

- Combined organ transplant
- Revision surgery after liver transplantation
- Previous liver transplantation
- Patients with a history of previous median laparotomy or transverse upper laparotomy, with a scar length of more than 15 cm
- Pre-existing abdominal hernia, except umbilical
- Any signs of inflammation on the abdominal wall before transplantation

### Endpoint

#### Primary endpoint

The primary endpoint of the study is the comparison of the time to incisional hernia over a period of 12 months between the two groups of patients with and without prophylactic mesh placement. Definition of incisional hernia: any detectable defect in the abdominal wall with intra-abdominal contents protruding beyond the aponeurosis [21].

#### Secondary endpoint

- Time to incisional hernia over a period of 24 months between the two intervention groups
- Rate of complications related to mesh placement, e.g., hematoma, seroma, chronic pain, wound dehiscence, mesh infections, mesh removal

#### Further parameters

- Date of birth
- Child-Pugh score and MELD score prior to liver transplantation
- Immunosuppression used
- Body Mass Index (BMI), height and weight
- Anamnesis of nicotine
- Diabetes mellitus
- Hepatocellular carcinoma
- Systemic corticosteroid therapy prior to transplantation
- Prior surgery of abdomen
- American Society of Anesthesiologists (ASA) classification

#### Trial interventions

This is a randomized controlled trial comparing the incidence of incisional hernia between patients undergoing liver transplantation with or without prophylactic mesh placement. The randomization is done using the “Randomizer for clinical trials” from the Institute for Medical Informatics, Statistics and Documentation, Medical University of Graz, Austria . A 1:1 randomization will be performed and patients will be assigned with a randomization number.

### Patient education and study enrollment

**Fig. 1:**
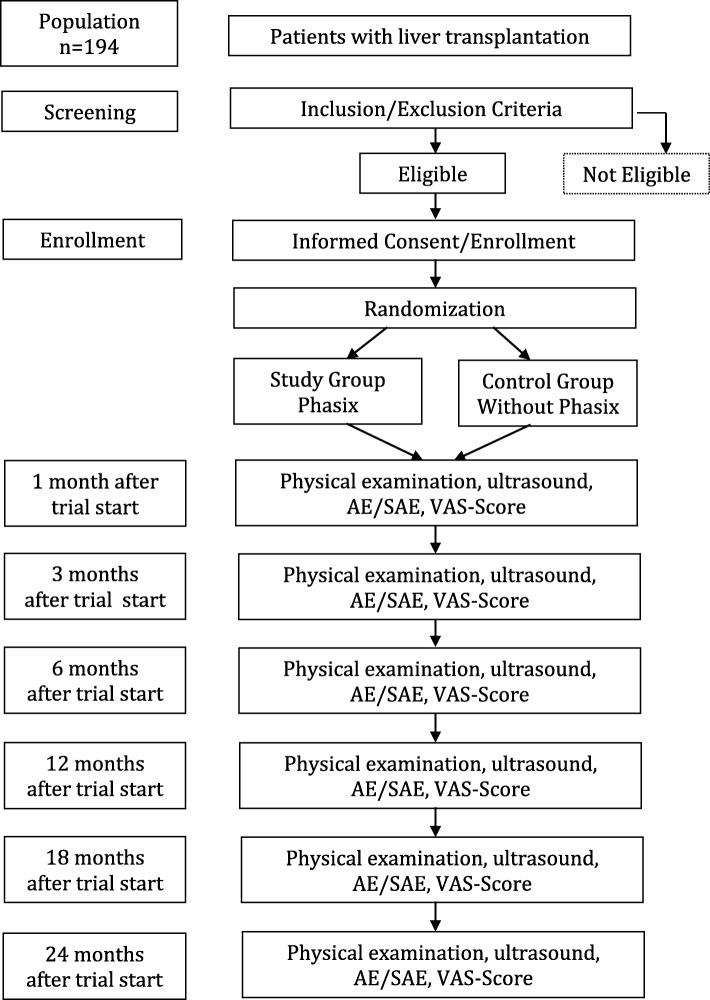
Standard Protocol Items: Recommendations for Interventional Trials (SPIRIT) Figure. Flow chart of the PRevention of INCisional hernia after liver transplantation (PRINC) trial. *AE* adverse event, *SAE* serious adverse event, *VAS score*, Visual Analogue Scale score for pain

**Fig. 2:**
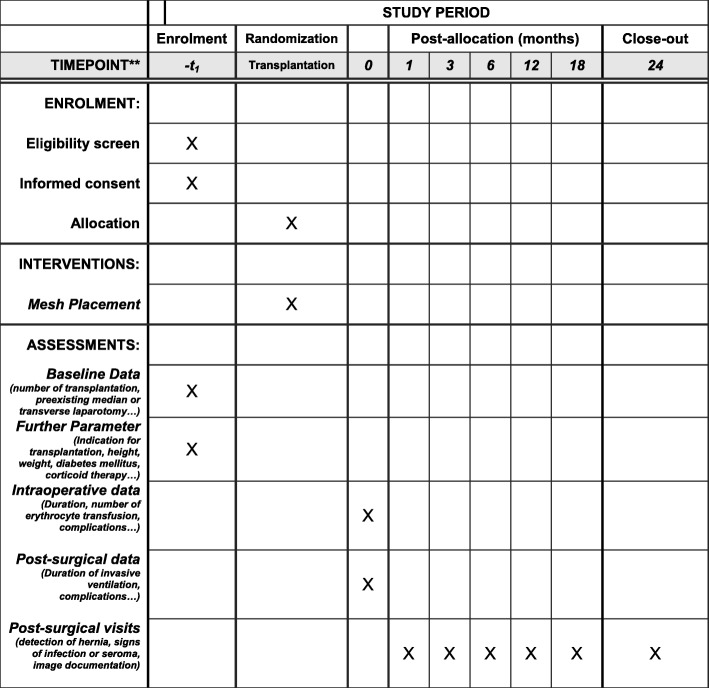
Template of content for the schedule of enrollment, interventions and assessments (Standard Protocol Items: Recommendations for Interventional Trials. (SPIRIT) Figure

As soon as a patient is listed for liver transplantation and no exclusion criteria apply, the patient will be informed about the study (Figs. 1 and 2, Additional file 1 Standard Protocol Items: Recommendations for Interventional Trials (SPIRIT) Figure). When a patient signs the written informed consent, they are enrolled in the study. Afterwards the initial condition of the patient will be assessed and the case report form (CRF) will be filled in. The status of the abdominal wall and the existence of risk factors for development of hernia after liver transplantation will be evaluated (see “Section 4.3”). The patients will be randomly allocated to either the standard of care or the intervention group. To reach target sample size, all patients who will receive a liver transplantation at our center are in ambulant treatment before our study team makes contact with them and comprehensive participant enrollment can then be performed.

### Surgical technique

Surgical access is through a midline laparotomy with right lateral extension. Liver transplantation is done using a standard or piggyback technique.

#### Abdominal-wall closure technique – Standard group

The closure of the J-shaped incision is started by placing two to three sutures of Ethibond 2–0 single stitches at the cranial end of the median part of the laparotomy and the far lateral end of the transverse part at a distance of 5–10 mm [22].

For the median laparotomy a full-thickness closure is performed using a PDS 2–0 double loop with intermittent single stitches using Ethibond 2 for augmentation (3–4 cm distance). The posterior sheath of the transverse incision is then closed by a PDS 2–0 double loop, followed by a third PDS 2–0 double loop closing the anterior sheath of the transverse incision. The transverse incision suturing is augmented by intermittently placing Ethibond 2 single sutures (approximately 5-cm distance).

The wound is closed using vicryl 4–0 single stitches for the subcutis every 1–2 cm. Skin closure is performed using staples.

#### Abdominal-wall closure technique – Intervention group

After closure of the fascia in the above fashion a Phasix™ (Bard – Davol Inc., Warwick, RI, USA) mesh is positioned on the anterior rectus sheath with an overlap of 3 cm along the total length and width. Mesh fixation will be done using PDS 3–0 in simple interrupted sutures each at a 5-cm distance along the margin of the mesh. A subcutaneous drainage will be used. For safety reasons the mesh is only implanted by experienced surgeons who have been instructed exactly on how to use it. Complications are handled according to the standards of our department.

### Postinterventional screening

#### Intraoperative data

Duration of operation.

Number of platelet transfusion, erythrocyte transfusion and plasma transfusion.

Operative complications (injuries to the intestine, bleeding).

#### Post-surgical data

Duration of invasive ventilation, complications, pulmonary infection, number of postoperatively required units of blood, days to first postoperative stool.

Postoperative complications: wound infection (according to the “New CDC Guideline for the Prevention of Surgical Site Infection”) [23], hematoma needing surgical revision, total subcutaneous drainage flow, seroma after removal of drainage, revision surgery.

#### Post-surgical visits

Post-surgical visits will take place 1, 3, 6, 12, 18 and 24 months after liver transplantation and include the following specific examinations:

- Ultrasound examination: detection of clinically non-detectable hernia
- Signs of infection of the surgical site
- Signs of seroma
- Visual Analog Scale (VAS) score
- Image documentation

#### Data management and statistical analysis

Almost all patients after liver transplantation will receive their post-transplant follow-up in our center; therefore, patients will come for a routine check-up and at the same time this is the follow-up for the study. The data for patients will be collected until 24 months after liver transplantation or until they drop out (revision surgery, death, withdrawal, loss to follow-up). We will use a data monitoring committee (DMC).

#### Planned statistics

The primary analysis will be for the intention-to-treat (ITT) population including all patients randomized and having had the liver transplantation performed. A per-protocol analysis with all patients without major protocol violations will also be performed.

Demographic and clinical parameters will be displayed with mean and standard deviation and median, minimum and maximum for continuous variables. Categorical variables will be shown via absolute and relative frequency. Furthermore, differences between the groups are calculated using Student’s *T* test or the Mann Whitney *U* test and the chi-squared test. The primary endpoint, time to incisional hernia over a period of 12 months, will be compared using a log-rank test. Additionally, we will also be plotting Kaplan-Meier survival curves by group looking at time to hernia development.

For sensitivity analysis a Cox regression analysis adjusted for age and MELD score will further be performed. Subgroup analyses for immunosuppression therapy (everolimus)-specific differences are planned. A *p* value < 0.05 will be statistically significant. We perform our analyses with SPSS v25.0 (SPSS Inc., Chicago, IL, USA).

#### Sample size estimation

We estimate from the current data that about 20% of patients will develop an incisional hernia in the standard group 1 year after surgery. In the intervention group in which we use Phasix™ (Bard – Davol Inc., Warwick, RI, USA) the prevalence of incisional hernia should drop to 5%.

Considering these assumptions and a level of significance of 5% and a power of 80%, we need to include 81 patients per group using a log-rank test. With regard to a drop-out rate of 10%, 178 patients need be included in the study.

#### Privacy

All included patients are coded with continuous numbers (pseudonymization). The data to be evaluated are saved at the Department of Surgery, Division of General Surgery and Division of Transplantation Surgery and only labeled with this number, using an Excel sheet data base on a computer with access restriction, and then assessed. Only authorized persons have access to the original data. Data will be stored for 15 years. Plans for investigators and the sponsor to communicate trial results to participants and healthcare professionals include publications and participation at scientific meetings.

#### Quality assurance

The principles of the International Conference on Harmonization-Good Clinical Practice (ICH-GCP) guidelines [23], the regulations of the Declaration of Helsinki [24] and local legal and regulatory requirements will be adhered to at all times during the trial period. Important protocol modifications will be managed according to the appropriate guidelines. Ancillary care or compensation to those who suffer harm from trial participation is covered by study-related insurance.

## Discussion

Due to a high incidence of incisional hernia after liver transplantation, patients would profit to a great extent from hernia prevention. Borab et al. [14] showed the benefits of prophylactic mesh placement in elective midline laparotomy, lowering the risk for the need for a second operation due to reducing the incidence of developing an incisional hernia. From our perspective there is a high variance for the incidence of developing an incisional hernia after liver transplantation. This variance is due to the different types of incisions used for liver transplant surgery. The transverse incision with subcostal extension has a high risk for developing an incisional hernia. J-shaped incisions seem to be associated with a lower risk but are still unsatisfactory. Placing a long-term resorbable mesh in an onlay position might improve the stability of the tissue and reduce the forces on the scar during the healing process until the mechanical strength of the resorbable mesh is resolved after 12 months and the scar tissue is stable. From our point view we hypothesize that this technique will reduce the incidence of developing incisional hernia from an estimated 20% to 5%.

For placement of the mesh the fascia needs to be dissected from the fatty tissue; this surgical step is associated with a minimally elevated risk for developing seroma, while the risk for surgical-site infection is reduced by the precise wound-closure technique and the choice of mesh material. The risk for the included patients is very low. The potential risks are wound-healing disorder, seroma, hematoma and mesh infection; all of which are evenly associated with the standard procedure.

### Trial status

Recruitment started on 1 January 2018. The protocol version of the study protocol is 3.0 from 18 April 2017; the protocol version of this manuscript is 1.4, 7 October 2018. Recruitment will be approximately completed in December 2021.

## Additional file

Additional file 1: Standard Protocol Items: Recommendations for Interventional Trials (SPIRIT) 2013 Checklist: recommended items to address in a clinical trial protocol and related documents*. (DOC 121 kb)

## Data Availability

There is no data available yet; patient enrollment started in January 2018.
